# Investigation of the properties of nanostructured Li-doped NiO films using the modified spray pyrolysis method

**DOI:** 10.1186/1556-276X-8-33

**Published:** 2013-01-18

**Authors:** Wu Chia-Ching, Yang Cheng-Fu

**Affiliations:** 1 Department of Electronic Engineering, Kao Yuan University, Kaohsiung, 82151, Taiwan; 2 Department of Chemical and Materials Engineering, National University of Kaohsiung, Kaohsiung, 81148, Taiwan

**Keywords:** Modified spray pyrolysis method, Nickel oxide, Lithium, Conductivity, Transparency

## Abstract

The lithium-doped nickel oxide (L-NiO) films were synthetized using the modified spray pyrolysis method with a two-step grown process. By observing the spectra of X-ray photoemission spectroscopy of L-NiO films, the intensity of Ni 2*p*_3/2_ peak of Ni^3+^ bonding state increases with increasing Li concentration that causes the decrease of transparency and resistivity. The L-NiO films with optimum characteristics were obtained at Li = 8 at%, where a p-type resistivity of 4.1 × 10^−1^ Ω cm and optical transparency above 76% in the visible region are achieved.

## Background

N-type transparent conductive oxide (TCO) films, such as indium tin oxide, aluminum zinc oxide, indium gallium zinc oxide, etc., are widely used as transparent electrodes, solar cells, and touch panels. However, not many TCO films have the p-type properties, and they are also required in other applications. Nickel oxide (NiO) films are a promising candidate for p-type semi-TCO in the visible light with the band gap (*E*_g_) values from 3.6 to 4.0 eV. NiO films have a wide range of applications, such as (1) transparent conductive films [1], (2) electrochromic display devices [2], (3) anode material in organic light emitting diodes [3], and (4) functional layer material for chemical sensors [4].

In the past, NiO films were prepared by various methods, including electron beam evaporation, chemical deposition, atomic layer deposition, sol–gel, and spray pyrolysis method (SPM) [5]. Sputtering is one of the most popular methods to deposit NiO films with low resistivity of 1.4 × 10^−1^ Ω cm [6]. The SPM is a very important non-vacuum deposition method to fabricate TCO films because it is a relatively simple and inexpensive non-vacuum deposition method for large-area coating. However, the resistivity of SPM deposited doped NiO films is about 1 Ω cm [7], which is almost 1 order of magnitude higher than that of sputter-deposited NiO thin films.

Undoped NiO has a wide *E*_g_ value and exhibits low p-type conductivity. The conduction mechanism of NiO films is primarily determined by holes generated from nickel vacancies, oxygen interstitial atoms, and used dopant. The resistivity of NiO-based films can be decreased by doping with lithium (Li) [8]. In 2003, Ohta et al. fabricated an ultraviolet detector based on lithium-doped NiO (L-NiO) and ZnO films [9]. However, only few efforts have been made to systematically investigate the effects of deposition parameters and Li concentration on the electrical and physical properties of SPM deposited NiO films. In this research, a modified SPM method was used to develop the L-NiO films with higher electrical conductivity. We would investigate the effects of Li concentration on the physical, optical, and electrical properties of NiO thin films.

## Methods

Lithium-doped nickel oxide films were prepared by SPM with 1 M solution. The nickel nitrate (Alfa Aesar, MA, USA) and lithium nitrate (J. T. Baker, NJ, USA) were mixed with deionized water to form the 2 to 10 at% L-NiO solutions. The isopropyl alcohol was added in L-NiO solution to reduce the surface tension on glass substrate; then, the solution was deposited on the Corning Eagle XG glass substrates (Corning Incorporated, NY, USA). The L-NiO films were then backed at 140°C and annealed at 600°C for densification and crystallization. The L-NiO films were formed according to the following reaction:

(1) $$\text{Ni}{\left(NO_{3}\right)}_{2}\cdot 6H_{2}O_{\Rightarrow }^{\text{Heated}}\text{NiO}+2NO_{2}+\frac{1}{2}O_{2}+6H_{2}O\text{,}$$

and the reaction of Li_2_O is

(2) $$2\text{LiN}O_{3}{}_{\Rightarrow }^{\text{Heated}}Li_{2}O+2NO_{2}+\frac{1}{2}O_{2}$$

The surface morphology and crystalline phase of L-NiO films were examined using the field-emission scanning electron microscope (FE-SEM) and X-ray diffraction (XRD) pattern, respectively. The atomic bonding state of L-NiO films was analyzed using the X-ray photoemission spectroscopy (XPS). The electrical resistivity and the Hall effect coefficients were measured using a Bio-Rad Hall set-up (Bio-Rad Laboratories, Inc., CA, USA). To determine the optical transmission and *E*_g_ of L-NiO thin films, the transmittance spectrum was carried out from 230 to 1,100 nm using a Hitachi 330 spectrophotometer (Hitachi, Ltd., Tokyo, Japan). The *E*_g_ value of L-NiO films was obtained from the extrapolation of linear part of the (*α*h*v*)^2^ curves versus photon energy (h*v*) using the following equation:

(3) $$\alpha hv=A\times {\left(hv-Eg\right)}^{n}\text{,}$$

where *α* is the absorption coefficient, h*v* is the photon energy, *A* is a constant, *E*_g_ is the energy band gap (eV), and *n* is the type of energy band gap. The NiO films are an indirect transition material, and *n* is set to 2 [10].

## Results and discussion

**Figure 1:**
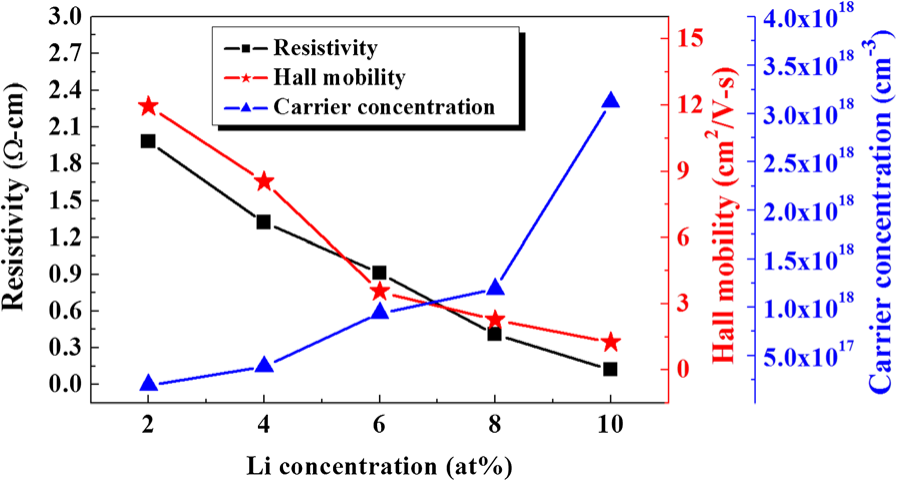
Resistivity, mobility, and carrier concentration of L-NiO films as a function of Li concentration.

Figure 1 shows resistivity (*ρ*), carrier mobility (*μ*), and carrier concentration (*n*) of L-NiO films as a function of Li concentration. As shown in Figure 1, the carrier mobility of L-NiO films decreases from 11.96 to 1.25 cm^2^/V/s as the Li concentration increases from 2 to 10 at%. For the carrier mobility, dopant materials as the scattering center, the carrier mobility will encounter more hindered concentration with increasing Li amount, which leads the decrease of mobility. The mobility of L-NiO films decreases with Li concentration; two reasons will cause this result: (1) As Li concentration increases, the number of Li atoms substituting the Ni atoms increases; thus, the carrier concentration increases from 1.91 × 10^17^ to 3.12 × 10^18^ cm^−3^. (2) As the Li concentration increases, more Li ions substitute Ni^2+^ in the normal crystal sites and create holes, as shown in Equation 4. Therefore, the resistivity of Li-doped NiO film with 2 at% doping amount is 1.98 Ω cm, and it decreases with Li concentration and reaches a minimum value of 1.2 × 10^−1^ Ω cm at the Li concentration of 10 at %.

(4) $$\overset{1}{\underset{2}{-}}O_{2}^{\left(g\right)}+Li_{2}O\Leftrightarrow 2O_{x}^{O}+2Li_{\text{Ni}\prime }+2\dot{h}$$

**Figure 2:**
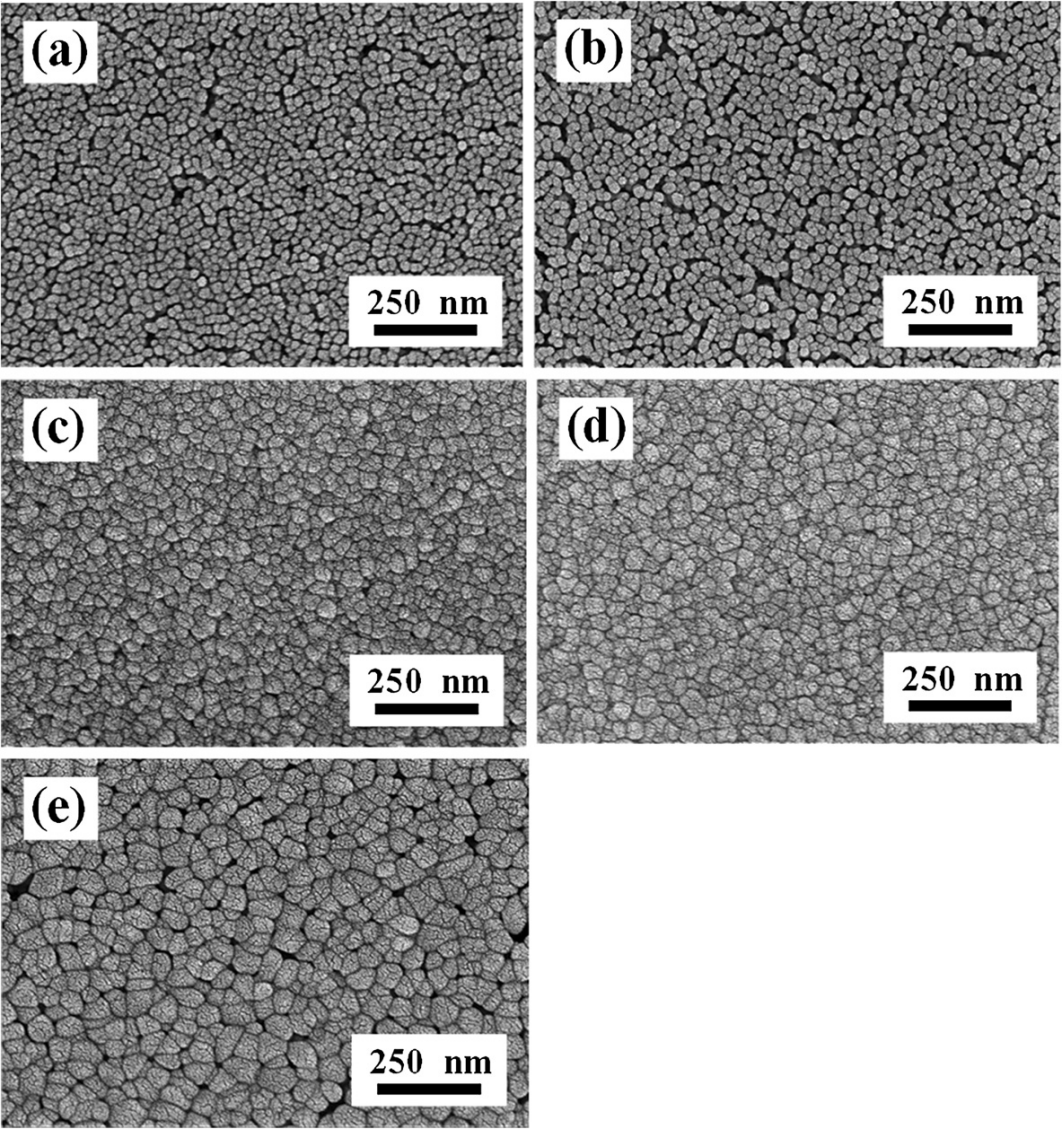
**Surface FE-EM images of L-NiO films with different Li concentrations.** (**a**) 2, (**b**) 4 (**c**) 6 (**d**) 8, and (**e**) 10 at %.

Figure 2 shows the surface FE-SEM images of L-NiO films. As Li = 2 at%, the L-NiO films have smooth but not compact surface morphology, and an average grain size of about 25 nm. The grain size of L-NiO films increases, and the pores decrease with increasing Li concentration. The improved grain growth can be attributed to the small radius, low activation energy, and high ionic mobility of the Li ions. During the crystal growth process, it is easier for these ions with low activation energy to escape from trap sites and transfer to nucleation sites, leading to larger grain size [11]. Therefore, the crystallization of the modified SPM deposited L-NiO films is better than that of traditionally SPM deposited films [7] and similar to that of sputter-deposited films [12]. The traditional method is to spray the nickel nitrate solution onto the preheated glass substrates (>300°C), which undergoes evaporation, solute precipitation, and pyrolytic decomposition. However, as the substrates are heated at higher temperatures, the evaporation ratio of solutions on glass substrate is too swift, resulting in the formation inferior to NiO films. In this study, using the modified SPM, the water and solvent in L-NiO solution were evaporated at 140°C, and the crystal growth of L-NiO films was formed at 600°C. Therefore, the better crystallization of L-NiO films is obtained using the modified SPM method.

**Figure 3:**
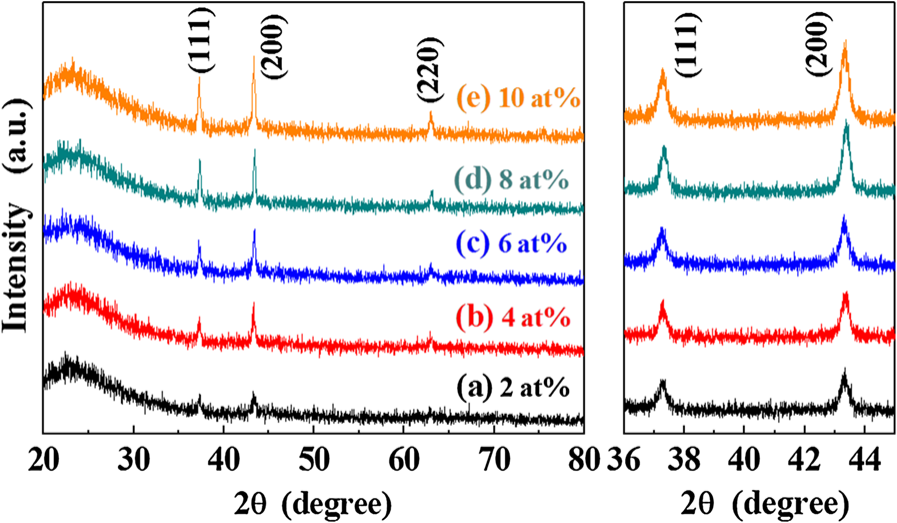
XRD and GIXRD patterns of L-NiO films as a function of Li concentration.

The XRD patterns of L-NiO films as a function of Li concentration are shown in Figure 3. All the L-NiO films have the polycrystalline structure and include the (111), (200), and (220) diffraction peaks. The diffraction intensity of (111), (200), and (220) peaks increases with Li concentration, which leads to the increase of crystallization. The grazing incidence angle X-ray diffraction (GIAXRD) patterns of L-NiO films in the 2*θ* range of 36° to 45° are also shown in the right side of Figure 3. Using the texture coefficient (TC) equation, each peak area in the GIAXRD spectra can be defined as:

(5) $$TC_{\left(\text{hkl}\right)}=\frac{I_{\left(\text{hkl}\right)}}{\sum I_{\left(\text{hkl}\right)}}\times 100\text{,}$$

where *h*, *k*, and *l* are the Miller indices, TC_(*hkl*)_ is the TC value of specific (*hkl*) plane, *I*_(*hkl*)_ is the measured peak intensity, and *ΣI*_(*hkl*)_ is the summation of all intensities for the peaks of L-NiO films. The TC_(111)_ value decreases from 0.394 to 0.357 as Li concentration increases from 2 to 10 at%. Conversely, the TC_(200)_ value changes from 0.602 to 0.641, while the TC_(220)_ value decreases from 0.393 to 0.360. It is well known that the (200) plane of ionic rock salt materials is considered as a non-polar cleavage plane and is thermodynamically stable, and the most stable NiO termination has a surface energy of 1.74 Jm^−2^. In contrast, the (111) plane is polar and unstable. Therefore, the (200) preferred orientation of L-NiO films can take on the better conductive properties and can resist electrical aging. In addition, the 2*θ* value of (111) diffraction peak is shifted from 37.22° to 37.38° as Li content increases from 2 to 10 at %. It implies that the Li^+^ (0.6 Å) ions substitute the Ni^2+^ (0.69 Å) ions, and the smaller radius of Li^+^ ions would result in a decrease of lattice constant.

**Figure 4:**
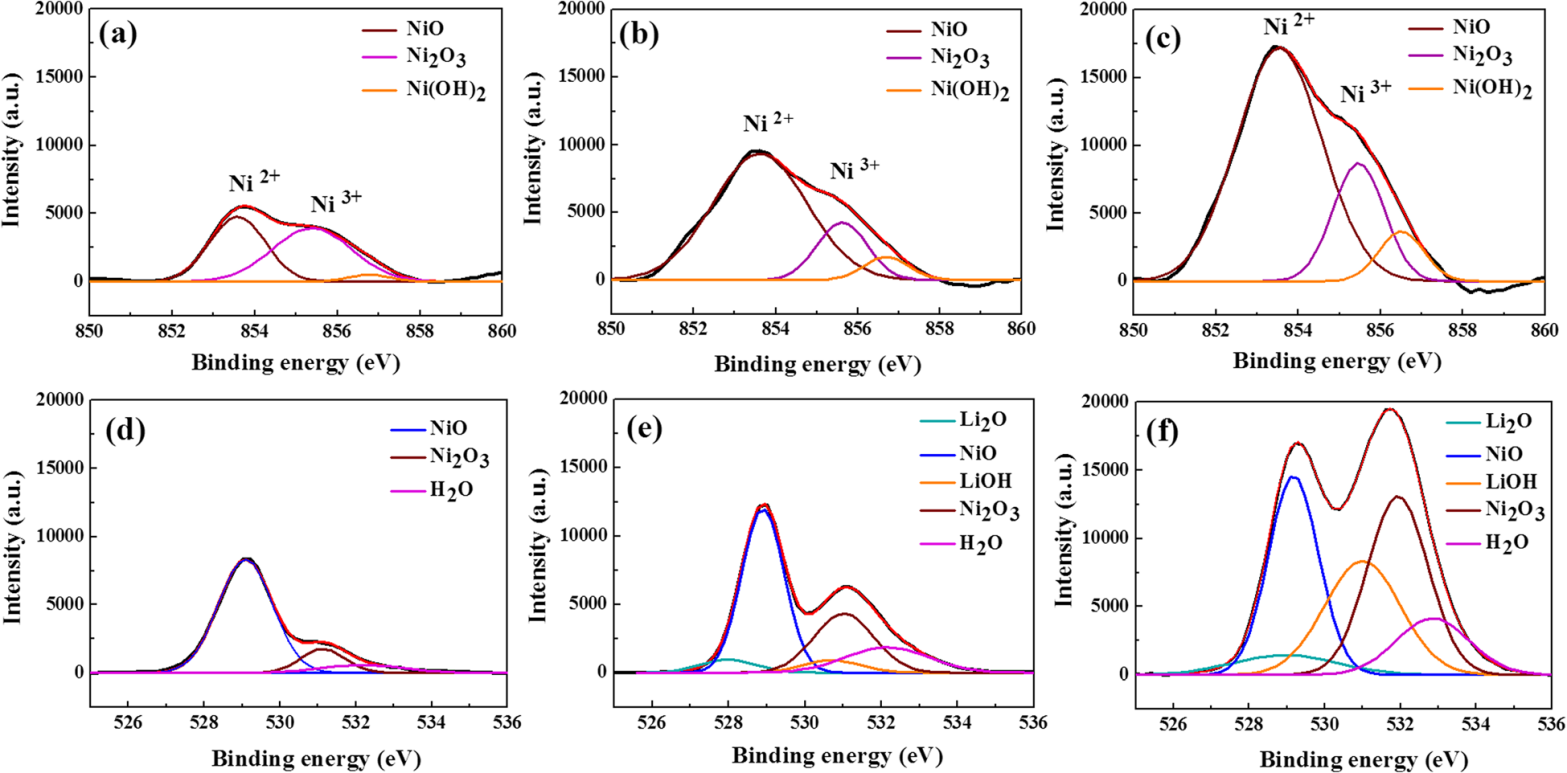
**Deconvolution of Ni 2*p***_**3/2 **_**and O 1*s *XPS spectra of L-NiO films.** Ni 2*p*_3/2_ XPS spectra of L-NiO films with (**a**) 2, (**b**) 6, and (c) 10 at% of Li. The O 1*s* XPS spectra of L-NiO films with (**d**) 2, (**e**) 6, and (**f**) 10 at% of Li.

The Ni 2*p*_3/2_ and O 1*s* XPS spectra of L-NiO films are shown in Figure 4 as a function of Li concentration. The deconvolution of Ni 2*p*_3/2_ electron binding energy to Gaussian fit for NiO, Ni_2_O_3_, and Ni(OH)_2_ peaks is 854.0, 855.8, and 856.5 eV, respectively [12,13]. For Ni 2*p*_3/2_ electron binding energy, the intensities of Ni^2+^ and Ni^3+^ bonding states increase with Li concentration and lead to the decrease of resistivity for the L-NiO films. The Ni(OH)_2_ bonding state is caused by the adsorption of H_2_O, and its intensity increases with Li concentration. The tendency of Ni 2*p*_3/2_ peak suggests that the Ni^3+^ bonding state increases with Li concentration, as shown in Figure 4a,b,c. The O 1*s* XPS spectrum of L-NiO films is shown in Figure 4d,e,f. The intensity of O 1*s* peak increases as Li concentration increases, and the deconvolution of electron binding energy of Li_2_O (528.5 eV), NiO (529.9 eV), LiOH (531.1 eV), Ni_2_O_3_ (531.9 eV), Ni(OH)_2_ (531.9 eV), and adsorbed O or H_2_O (532.5 eV) exists in the L-NiO films [13-17]. The intensity of LiOH bonding state, which is caused by the combining Li^+^ and the OH^−^ bonds of H_2_O, slightly increases with Li concentration. Compared with other electron binding energy, the binding energies for the Ni 2*p*_3/2_ of Ni(OH)_2_ (856.2 eV) and the O 1*s* of LiOH (531.1 eV) are weaker in the modified SPM deposited L-NiO films. This result demonstrates that the non-polar (200) phase of L-NiO films increases with Li concentration (as shown in Figure 3) because the non-polar (200) phase exists with fewer dangling bonds, which cause the less binding probability to exist between in L-NiO films and water molecules.

**Figure 5:**
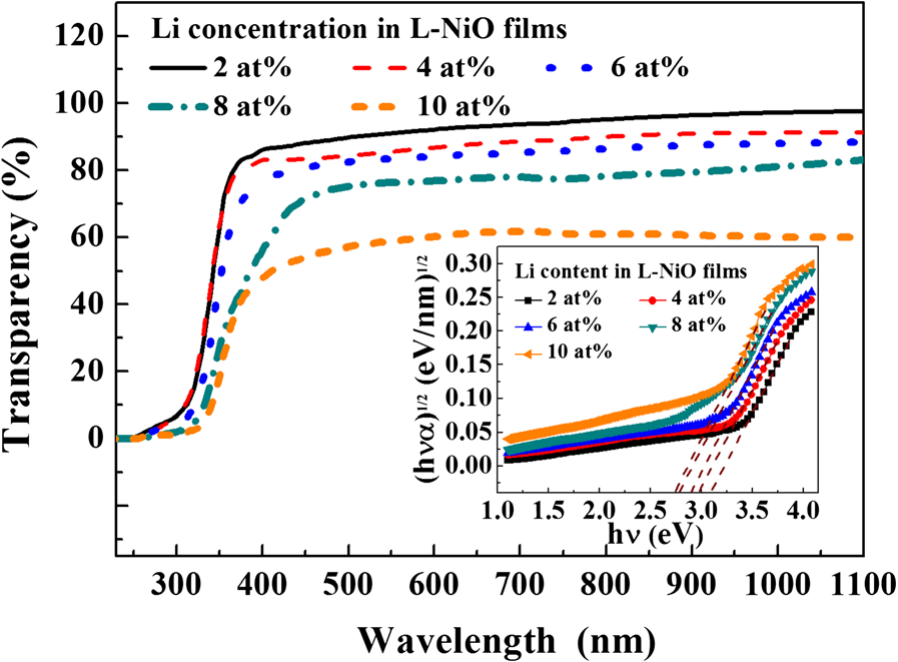
Transmittance spectra of L-NiO films deposited with different Li concentrations.

The optical transmittance spectra of L-NiO films in the wavelength range from 200 to 1,100 nm are shown in Figure 5. The transparency of L-NiO films decreases from approximately 89% to approximately 57% as Li concentration increases from 2 to 10 at%. Two reasons will cause this result: (1) Observing from the surface morphology (FE-SEM images), the crystallization and grain size of L-NiO films increase with Li concentration, and the scattering effect occurs in higher Li-doped concentration. (2) The existence of Ni^3+^ ions measured from XPS gives rise to the brown or black colorations [18]. The inset of Figure 5 presents the plots of (*α*h*ν*)^1/2^ versus h*ν* (photon energy) for L-NiO films. The optical band gap has been calculated by extrapolating the linear part of the curves. The optical band gap of L-NiO films gradually decreases from 3.08 to 2.75 eV with Li concentration because of the decrease in carrier mobility. These results are caused by the dopant Li ions which act as the scattering center and hinder the carrier to move.

## Conclusions

Non-vacuum SPM method was used to deposit high quality p-type L-NiO films. The (200) preferred orientation of L-NiO films increases over (111) as the Li concentration increases, which would cause the better conductive properties and resist electrical aging in the L-NiO films. In this study, the characteristics of modified SPM deposited L-NiO films were comparable to the sputter-deposited ones, and the optimum Li doping amount is set at 8 at %.

## Competing interests

The authors declare that they have no competing interests.

## Authors’ contributions

C-CW participated in the fabrication of Li doped NiO films, SEM, XRD and XPS analysis. C-FY participated in the Hall measurement and calculated the optical band gap of L-NiO. All authors read and approved the final manuscript.

## Authors’ information

C-CW was born in Taiwan, in 1979. He received the Ph.D. degree in electrical engineering from the National Sun Yat-sen University, Kaohsiung, Taiwan, in 2009. In 2009, he joined department of electronic engineering, Kao Yuan University, where he investigated on organic/inorganic nanocomposites materials, integrated passive devices (IPDs), transparent conductive oxide (TCO) films, electron ceramics and carbon nanotubes and graphene.

C-FY was born in Taiwan, in 1964. He received the BS, MS, and Ph.D degree in electrical engineering from the National Cheng Kung University, Tainan, Taiwan, in 1986, 1988, and 1993. In 2014, he joined department of Chemical and Materials Engineering, National University of Kaohsiung, where he investigated on ferroelectric ceramics and thin films, application ferroelectric materials in memory devices, organic/nanotubes nanocomposites, organic/inorganic nanocomposites, YZO thin films, transparent conduction oxide thin films and their applications in solar cells, microwave antennas, and microwave filters.
